# An Adaptive Antiretroviral Therapy Adherence Intervention for Youth with HIV Through Text Message and Cell Phone Support with and without Incentives: A Sequential Multiple Assignment Randomized Trial (SMART)

**DOI:** 10.1007/s10461-024-04558-x

**Published:** 2024-12-20

**Authors:** Marvin E. Belzer, Karen MacDonell, Demetria Cain, Samiran Ghosh, Richard Zhao, Julie McAvoy-Banerjea, Sitaji Gurung, Sylvie Naar

**Affiliations:** 1 Children’s Hospital of Los Angeles, Los Angeles, CA USA; 2 Center for Translational Behavioral Science, Florida State University, Tallahassee, FL USA; 3 Department of Psychology, Hunter College, City University of New York, New York, NY USA; 4 Department of Biostatistics and Data Science, University of Texas School of Public Health, Houston, TX USA; 5 Department of Health Sciences, New York College of Technology, City University of New York, New York, NY USA

**Keywords:** HIV, Medication adherence, Adolescents, RCT

## Abstract

Youth living with HIV have low rates of medication adherence. Youth ages 15–24 years with adherence ≤ 80% or with HIV RNA PCRs (VL) ≥ 200 recruited through social media and clinical sites were randomized to brief weekday cell phone support (CPS) calls or daily, two-way, personalized text message (SMS) reminders for 3 months. Those with VL ≥ 200 or adherence ≤ 80% were rerandomized to receive SMS or CPS with monthly incentives for those utilizing the intervention at least 75% of days for 3 months. Those with VL < 200 or adherence > 80% after the initial 3 months were rerandomized to usual care or 3 months of tapered, 2x/week CPS or SMS. Self-reported adherence and VLs were collected every 3 months for one year. Eighty-three youth were recruited with 81% identifying as cisgender males, 55% Black, 22% Latine/x, and 76% gay, and 56% recruited from the Southern US. Both cohorts initially randomized to CPS and SMS demonstrated significant improvements in adherence over the 12-months (*P* <.001). Participants randomized to CPS had significant improvements in 7-day self-reported adherence over 12 months compared to those on SMS (*P* <.027). Those receiving a tapered intervention for an additional 3 months had improved self-reported adherence compared to those randomized to the standard of care arm (*P* <.001). Both SMS and CPS appear to be effective interventions for youth with poor antiretroviral adherence. Tapering the intervention for an additional 3 months is useful in maintaining adherence after the initial intervention. Additional research is required to determine how to best sequence these interventions, including the use of incentives.

**Graphical Abstract:**

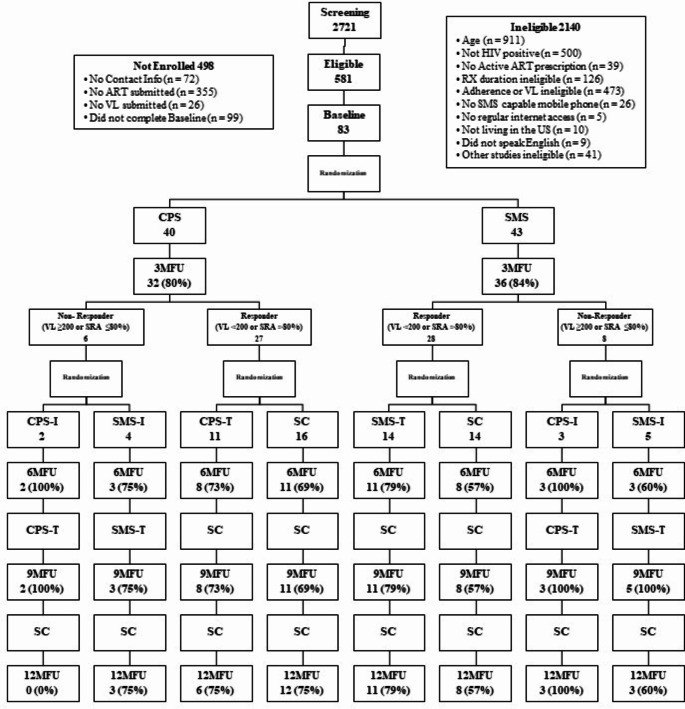

## Introduction

Youth with HIV (YWH) frequently struggle to adhere to antiretroviral therapy (ART) [1, 2]. There have been considerable improvements with ARTs, specifically integrase inhibitors. They have improved tolerance and potency, allowing for a reduction in overall adherence required to reach viral suppression. Despite this, youth continue to have the lowest rates of viral suppression when compared to other age groups [3, 4]. One reason for this is that adherence to ART tends to be poor among adolescents and emerging adults. ART adherence is critical to the long-term health of YWH and in reducing the risk of HIV transmission to sexual partners. The CDC promotes complete viral suppression as the ultimate tool for *Ending the HIV Epidemic in the US*, and persons who maintain an undetectable HIV viral load cannot sexually transmit the virus to others (U = U; Undetectable = Untransmittable) [5]. More youth-specific, evidence-based interventions to improve ART adherence are critically needed. In addition, no single intervention is likely to be both acceptable and effective for all populations. The toolbox for clinicians must be broad, and interventions need to be adaptable based on clinic- or youth-specific needs.

### Current ART Interventions for Youth

The CDC currently lists 29 Medication Adherence Evidence-based Behavioral Interventions [6]. Three of these are youth specific including *Project Yes!* [7], *Text Messaging* [8], and *Project nGage* [9]. *Project Yes!* was an HIV clinic-based peer mentoring program for youth ages 15–24 in sub-Saharan Africa, and 72% of the participants acquired HIV through perinatal transmission. There were significant improvements in viral load suppression at 6 months in the intervention group [7]. The *Text Messaging* intervention studied two-way text messaging in youth ages 16–29 from Chicago, which demonstrated significant improvements with greater than 90% self-reported adherence at both 3 and 6 months. Viral load suppression was not reported [8]. *Project nGage* was a social network support intervention designed to improve retention in care and ART adherence in young Black men ages 16–29 who have sex with men. It involved an initial 90-minute session with the individual and a support confidant followed by 4 telephone boosters at 2, 5, 8, and 11 months. Results showed the intervention group had significantly more > 90% self-reported adherence at 12 months compared to the control group. There was no difference noted in viral load suppression [9].

Other youth-specific adherence interventions with some evidence of efficacy include cell phone support (CPS) [10], motivational interviewing [11], directly observed therapy [12], and multisystemic therapy [13]. CPS is a brief, mobile intervention that can be delivered using a centralized delivery model, which may decrease cost and increase scalability and sustainability. A prior CPS pilot study of 37 youth with severe nonadherence demonstrated significant improvements in self-reported adherence and reductions in viral load at both the end of the 24-week intervention and 24 weeks post intervention. This study utilized incentives to support intervention attendance, and still, many participants in the intervention group did not adhere to the intervention (thus acceptance was not universal) [10]. Lastly, a systematic review and meta-analysis of the effectiveness of mobile text message reminders to improve youth adherence in 10-19-year-olds reported 5/7 studies found improved adherence, and 2/7 did not. Overall, the meta-analysis did not demonstrate significant improvements in adherence. Only 2 of the 7 studies were conducted in the US [14].

A recent study utilizing coaching for youth nonadherent to antiretroviral therapy, Triggered Escalating Real-time Adherence (*TERA*), enrolled 88 participants ages 13–24 years with detectable viral loads. Participants were paired with a coach for 12 weeks, and coaches were trained in motivational interviewing. Coaches met through video links at baseline and 4 and 12 weeks and provided support as needed through text messaging and phone calls when electronic data monitoring (EDM) indicated medications were being taken late. The intervention improved adherence but not viral suppression at 12 weeks (35% in *TERA* participants and 36% in control participants achieved viral suppression). Adherence was measured through the median percentage of days opening the EDM and was 72% in the *TERA* arm and 41% in the standard of care arm during the 12 weeks following the intervention [15].

### Theoretical Base

This intervention is guided by the conceptual model of supportive accountability [16]. This model is described in our methods manuscript [20]. Supportive accountability was developed to guide research into human support components of mobile health interventions. The underlying premise is that human support from a coach who is viewed as trustworthy, knowledgeable, and benevolent increases adherence through accountability to that person.

This study utilized a Sequential Multiple Assignment Randomized Trial (SMART) design, a design that can be used to inform the construction of adaptive interventions [17]. SMART is a within-participant adaptive, multi-stage longitudinal trial where randomization occurs at two time points: once at the beginning and again at an intermediate stage based on a pre-defined treatment decision (e.g., responder vs. non-responder). In addition to efficiency, another advantage of SMART over multiple trials is the involvement of the same participants in all phases of intervention testing, which increases methodological rigor. Results inform how initial and subsequent stage treatments work with (synergistically) or against (antagonistically) each other and inform hypotheses about moderators of sequenced treatments. SMART is used as a rigorous approach to intervention development (T1), and with larger sample sizes SMARTs can also be powered to test clinical trial hypotheses (T2) [18]. When conducted in the context of real-world settings with evidence-based strategies, SMART can be utilized in effectiveness-implementation research (T3/T4) research as in the this study [19]. In this study, the SMART design was used to address specific research questions around intervention dose and incentives, as described in the next section. It should be noted that the sample was significantly smaller than planned; thus, the SMART primary analysis was limited to the first randomization, and analyses around dose and incentives exploratory.

The aim of this study, *SMART*, was to test intervention sequences to develop an adaptive adherence intervention (SMART design), which utilizes several mHealth intervention strategies (text messaging vs. cell phone support with and without incentives and followed by tapering vs. no tapering) to promote adherence to ART and maintain viral load (VL) suppression in YWH from across the United States. Despite a smaller-than-anticipated sample size, the study retained the SMART design (e.g., randomization to one treatment option plus a second randomization based on pre-defined treatment response); however, the aims focused on incentives and tapering were shifted to exploratory. All interventions were delivered remotely, utilizing a central research center. Because the pilot found no improvements in the control arm (usual care) over a 12-month period, we did not feel it was ethical to include a control group. Instead, we decided to test our hypothesis that initially utilizing CPS would have a significant improvement in adherence compared to initially utilizing SMS intervention. In addition, this study aimed to increase understanding of the context for wide-scale implementation of this type of intervention as well as to understand the benefit of incentives for non-responders and tapering of interventions for responders.

## Methods

*SMART* utilized a sequential multiple assignment randomized trial (SMART) design where each stage corresponds to a critical treatment decision and randomization takes place at those two decision points. Nationwide enrollment for this adaptive medication adherence intervention was conducted between July 2018 and March 2021 (NCT#: NCT03535337). Participants were recruited at baseline, randomized to receive either CPS or SMS for medication adherence, and completed follow-up assessments at 3-, 6-, 9-, and 12-months post-baseline. Detailed methods are discussed in our protocol manuscript [20]. Participants were enrolled from across the United States. This study was executed remotely from Hunter College, City University of New York, where all phone calls and text messages originated.

### Participants

Participants were deemed eligible to participate if they were: [1] 15 to 24 years of age; (2) living with HIV; (3) prescribed ART medication for a minimum of 3 months; (4) willing to provide proof of viral load ≥ 200 copies/mL within 12 months prior to baseline enrollment (self-submission, obtained from a medical provider, or through providing a specimen at a Quest Diagnostics laboratory) or self-report adherence (SRA) ≤ 80% in the past 4 weeks (SRA accepted after June 2020); (5) sole owner of a device capable of sending and receiving calls and text messages; (6) able to provide consent (i.e., HIPAA authorization or release of information form) for the research team to communicate with participant’s HIV care provider team about VL test results. Participants were excluded if (1) their mental, physical, or emotional capacity did not permit them to complete the protocol as written; (2) they were unable to understand written or spoken English; or (3) they were concurrently participating in any behavioral research intervention designed to impact medication or care adherence.

### Recruitment Procedures

Participants were recruited through paid advertisements on social media (e.g., Facebook, Instagram) and geo-social networking and dating apps. Potential participants clicked on ads that redirected them to a screening survey to determine eligibility. Further details related to the protocol, methods, and recruitment can be found in previously published articles [20, 21].

### Enrollment Procedures

Participants completed the study screener to be eligible, after which they received a unique Qualtrics link for submitting proof of ART regimen and HIV VL ≥ 200 copies/mL (if available) to determine final eligibility. Upon eligibility confirmation, the study team sent the potential participant the SMART enrollment link that included the study consent/assent form, HIPAA authorization form, and the baseline survey. Completion of all three portions confirmed participants as enrolled in SMART.

### Randomization

**Fig. 1:**
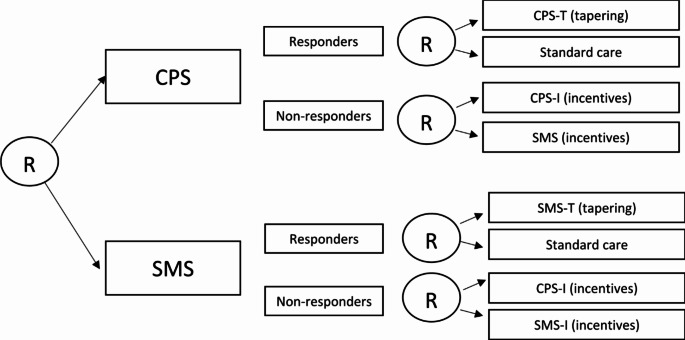
The SMART study adaptive treatment design. CPS: cell phone support. SMS: text messaging support. Randomization 1 occurred at baseline and randomization 2 at 3-month follow-up. Response was defined as viral load ≥ 200 copies/mL. At 6-month follow-up, non-responders (determined at randomization 2) continued to receive CPS or SMS, but with tapering. Responders remained in standard care. All participants shifted to standard care at 9-month follow-up

Participants underwent a first randomization at the end of their baseline assessment. Stratification was based on method of HIV transmission: perinatal vs. other. Participants were randomized into one of two interventions: (1) CPS or (2) SMS, each of which lasted three months. At the 3-month follow-up, a second stratified randomization occurred into one of eight possible intervention trajectories as follows (see Fig. 1):

- CPS responders (VL < 200 copies/mL or Self-Reported Adherence (SRA) > 80% when VL was unavailable after June 2020) were randomized to either 9 months of Standard Care (SC) or 3 months of Tapered CPS (CPS-T) followed by 6 months of SC;
- CPS non-responders (VL ≥ 200 copies/mL or SRA ≤ 80% when VL was unavailable after June 2020) were randomized to 3 months of Incentivized CPS (CPS-I) or Incentivized SMS (SMS-I) followed by 3 months of CPS-T or Tapered SMS (SMS-T), respectively, and then 3 months of SC for both groups thereafter;
- SMS responders (VL < 200 copies/mL or SRA > 80% when VL was unavailable after June 2020) were randomized to either 9 months of SC or 3 months of SMS-T followed by 6 months of SC; and.
- SMS non-responders (VL ≥ 200 copies/mL or SRA ≤ 80% when VL was unavailable after June 2020) were randomized to 3 months of CPS-I or SMS-I followed by 3 months of CPS-T or SMS-T, respectively, and then 3 months of SC for both groups thereafter.

Participants who were unable to provide a valid HIV VL result on their own at the 3-month follow-up had approximately 30 days to obtain a HIV VL performed at a local Quest Diagnostics laboratory or for a medical provider to release this information to the study team. In order for study staff to receive patient medical records and VL results from medical providers, participants completed and signed a Release of Information (ROI) form. Participants who were unable to provide this information within the timeframe of 30 days after their 3-month follow-up assessment were re-randomized as non-responders to an incentive arm (CPS-I or SMS-I).

Randomization criteria changed in June 2020, due to the challenges participants had in accessing VL during COVID restrictions. If a participant was unable to provide a VL, we utilized SRA > 80% to indicate whether a participant was a responder.

### Condition Description

#### Cell Phone Support (CPS)

Each participant randomized to CPS was assigned an adherence facilitator (AF) for their phone calls. During the initial CPS call, the AF and the participant mutually agreed upon a start date and call time that was after the daily ART dosage time. Calls from the AF occurred once every business day for three months and took typically less than five minutes. To protect the confidentiality of the participant, the AF confirmed the participant answered the phone via voice recognition and the optional use of a code word for identification purposes to further protect participants’ privacy.

On each call, AFs assessed if the participant took their medication for that day along with days when calls to participants were not completed (i.e., weekends and holidays). If the participant had not yet taken their ART medication(s), the AFs waited for the participant to retrieve and take their medication, if available, but the AF did not request that the participant take their medication during the call if their typical ART dose was at night to avoid double dosing in a single day. If the participant was non-adherent, the AFs assessed reasons for non-adherence and engaged the participant in brief problem solving around the identified barriers. The AFs also discussed any new or ongoing problems in the participant’s life (e.g., related to housing, transportation, or food), and provided support and problem-solving to address these issues.

If a participant did not answer a scheduled call, the AF left a reminder voicemail requesting that the participant call back within 30 min. If the participant did not return the call within 30 min, the AF repeated the call attempt. If the participant returned the phone call after more than 30 min have elapsed, the AF would still conduct the call.

#### Text Message Support (SMS)

Participants randomized to the SMS intervention received daily personalized SMS adherence reminders for 3 months. Participants chose the timing and wording of the text message. Participants were asked to text back if they did or did not take their ART medications. The texts were sent through Trumpia, a robust, customizable, and trackable SMS technology compliant with HIPAA laws as all personal identifiable data were encrypted.

#### Incentives and Tapering

After 3 months of either the CPS or SMS intervention, participants were sorted into new intervention arms depending on their VL or SRA from the 3-month survey if VL was unavailable (see Fig. 1). All CPS and SMS responders (VL < 200 copies/mL or SRA > 80%) were re-randomized into their respective tapered CPS or SMS intervention arm or into standard care. CPS-T and SMS-T lasted for 3 months until the 6-month follow-up, after which participants went into standard care. At this point, all responders were in standard care and continued throughout the 9- and 12-month follow-ups. All CPS and SMS non-responders (VL ≥ 200 copies/mL or SRA ≤ 80%) were re-randomized into CPS-I or SMS-I with the addition of a $50 incentive for responding to the intervention 75% of the time or more each month. This was implemented for 3 months until the 6-month follow-up, after which the intervention was tapered respectively to CPS-T or SMS-T two times per week. After the 9-month follow-up, participants entered standard care until completion of the study at the last 12-month follow-up.

All study procedures were approved by the Florida State University IRB.

#### Adherence Facilitators

The AFs for CPS were paraprofessionals knowledgeable about HIV treatment, skilled in interpersonal communication, and aware of the different types of local healthcare systems. All AFs had previously been trained in motivational interviewing and attended a two-hour training designed to familiarize AFs with the protocol, purpose of the study, definition of adherence, role of the AF, HIV basics, cultural humility, building rapport, effective communication, and legal issues. Following the training, AFs participated in a minimum of two telephone role plays during which they practiced the initial and daily phone calls. AFs attended monthly meetings for study personnel to augment training and support intervention fidelity. To this end, the quality assurance manager provided feedback to AFs based on their review of randomly selected digital recordings of phone calls to participants. This feedback was used to maintain high implementation fidelity and consistency in delivery across AFs.

#### Intervention Monitoring/Quality Control

Intervention phone calls between the AFs and participants in this study were digitally recorded and saved on a secure network for review. The quality assurance manager randomly selected and reviewed at least 20% of all audio files for each AF during the initial three months. If 90% of reviewed files were found to be adherent to the intervention checklist, then the quality assurance manager was required to review only 10% of all audio files after the initial three months; however, they often reviewed more than 10%. The recordings were assessed for adherence to the phone call script, centering of participants in discussion, order of content discussed, appropriate call length, and appropriate responses, advice, and referrals. The quality assurance manager shared any discrepancies and provided any critical feedback to the AFs as soon as possible. Other feedback was provided during the monthly AF meetings. This ongoing feedback process ensured the integrity of the intervention.

#### Retention and Follow-up

Follow-ups occurred 3-, 6-, 9-, and 12-month after enrollment, and participants completed a 30-minute online survey within 14 days before and 28 days after their target follow-up date. Once the follow-up window opened 2 weeks prior to the target follow-up date, study staff sent the unique survey and VL submission links to the participant to complete during the 6-week follow-up window. The team used automated reminders to communicate with participants about remembering to complete their surveys and VL testing. The study team utilized multiple contact methods for difficult-to-reach participants (e.g., alternate phone numbers, email, text messaging, family members, friends, and healthcare providers).

#### Incentives and Compensation

Participants received compensation up to $500 total for completing all study assessments or up to $650 total for completing all study assessments plus the incentivized intervention, if re-randomized so (Fig. 1). Participants received $60 at baseline after completing the online survey and providing the proof of viral load. A $10 increase in compensation was offered over time at each subsequent follow-up where participants completed the survey and submitted viral load results. The team also offered up to two $10 bonus payments for participants who completed the survey or submitted the viral load results within two weeks of the date they were sent out. Participants in the CPS-I and SMS-I interventions who reached the 75% monthly adherence to calls/text responses received $50 as part of the incentivized intervention arm. Compensation was provided via electronic Amazon gift cards.

## Measures

*Demographics* included race/ethnicity (African American/Black, Latino/a/x/e, White, Multiracial/Other), gender identity (cisgender man or woman, transgender man or woman, non-binary, or other), US region (Northeast, Midwest, South, West), employment status (yes, no), education (less than high school, high school or equivalent, some college or above), sexual identity (gay, heterosexual, other), and age.

*ART medication adherence* was assessed using a visual analog scale for the last 7 days and the last 30 days. For both time periods, participants were asked, “What percent of your prescribed HIV medication have you taken. 0% means you have taken no medication, 50% means you have taken half your medication, 100% means you have taken every single dose of your medication.” Participants self-reported their ART adherence in the past 7 days and in the past 30 days. Medication self-efficacy was the average score of three medication adherence questions asking about certainty to taking the right amount of medication at the right time. Responses were on a 5-point scale from *very sure I can* (5) to *very sure I cannot* (1).

*Depression* was assessed using Patient Health Questionnaire (PHQ-8), which was the sum of eight questions asking, “Over the past 2 weeks, how often have you been bothered by any of the following problems?” Different scenarios included, “Little interest or pleasure in doing things,” or “Feeling down, depressed or hopeless.” Responses were on a 4-point scale from *Not at all* (0) to *Nearly every day* (3).

*Patient Activation Measure* [22] was the score of 10 questions asking participants how much they agreed with statements regarding their role in their health care. Examples of questions included, “When all is said and done, I am the person who is responsible for taking care of my health,” and “Taking an active role in my own health care is the most important thing that affects my health.” Responses were on a 4-point scale from *Strongly Disagree* (1) to *Strongly Agree* (4) and scored by the scale’s developer.

*Substance use* was assessed using the Alcohol, Smoking and Substance Involvement Screening Test (ASSIST) [23]. A global risk score was created from questions assessing tobacco, alcohol, cannabis, cocaine, amphetamine, crystal methamphetamine, inhalants, sedatives, hallucinogens, and opioids use.

### Data Analyses

The data analyses were conducted on the de-identified data. Bivariate comparisons (t-tests and chi-squares) were conducted to examine the group difference (i.e., SMS vs. CPS), if any, at baseline. For the primary analysis, we considered two primary outcome variables namely adherence (7-day and 30-day recall) and viral load, and two initial interventions (SMS vs. CPS) are compared. A mixed-effect linear model was used, which can accommodate all data including intermittent as well as premature dropouts under the “ignorable” condition [24]. The model includes participant-specific random effects and an intervention-specific fixed effect. A random intercept and slope are also included. Our primary interest was the significance of the intervention*time interaction term, which if significant will indicate a differential course across two initial intervention arms. The model also includes some of the clinical and demographic variables as a covariate. The goodness-of-fit of the overall model is indicated by AIC as well as negative log-likelihood criteria.

## Results

### Sample

Eighty-three participants aged 16–24 years were recruited primarily through social media and at Adolescent Medicine Trials Network for HIV Interventions (ATN) sites from July 2018 to March 2021. Our consort diagram details recruitment and retention. A more detailed description of our recruitment has been published [21]. Forty were randomized at baseline to CPS and 43 to SMS. Mean age was 22 years, 81% cisgender men, 55% Black/African American and 22% Latine/x, 53% recruited from the Southern US, and 76% identified as gay (Table 1). Baseline PHQ-8 was 9.2 (SD 7.19) and Global Risk substance use was 22.3 (SD 15.56). Baseline Self-Reported Adherence 7-day was 69.38% (CPS 63.68% and SMS 74.52% *p* = .138), and Self-Reports Adherence 30-day was 71.66% (CPS 68.55% and SMS 74.33% *p* = .355). Baseline viral load was < 200 copies/mL in 58.8% of those assigned to CPS and 62.9% of SMS (recall participants were also eligible if SRA < 80%) (see Table 1).

**Table 1:** Sample characteristics by study group (*n*=83)

	Total	CPS	SMS	X^2^	*p* Value
	*n* (%)	*n* (%)	*n* (%)		
*n*	83 (100.0)	40 (48.2)	43 (51.8)		
Gender Identity					
Cisgender Man	67 (80.72)	32 (80)	35 (81.4)	0.45	0.93
Cisgender Woman	11 (13.25)	5 (12.50)	6 (13.95)		
Other	5 (6.02)	3 (7.50)	2 (4.66)		
Race and Ethnicity					
African American	44 (55.42)	20 (50)	24 (55.8)	0.53	0.913
Latinx/e	19 (21.69)	9 (22.5)	10 (23.3)		
White	7 (12.05)	4 (10)	3 (7.0)		
Multi-racial/ Other	13 (10.84)	7 (17.5)	6 (14.0)		
Region					
Northeast	11 (13.3)	3 (7.5)	8 (18.6)	4.12	2.49
Midwest	12 (14.5)	8 (20)	4 (9.3)		
South	44 (53)	20 (50)	24 (55.8)		
West	16 (19.3)	9 (22.5)	7 (16.3)		
Employment					
yes	44 (53)	24 (60)	20 (46.5)	1.51	0.219
Education					
Less than High School	13 (15.7)	7 (17.5)	6 (14)	1.04	0.594
High School or Equivalent	32 (38.6)	17 (42.5)	15 (34.9)		
Some College or Above	38 (45.8)	16 (40	22 (51.2)		
Sexual Identity					
Gay	63 (75.9)	29 (72.5)	34 (79.1)	0.49	0.484
Heterosexual or Other	20 (24.1)	11 (27.5)	9 (20.9)		
	*M(SD)*	*M(SD)*	*M(SD)*	*t*-test Statistic	*p* Value
Age	22.27 (1.78)	22.58 (1.63)	21.98 (1.88)	1.54	0.538
Substance Use - Global Risk	26.33 (15.56)	28.6 (15.49)	24.23 (15.5)	1.28	0.203
Mental Health - PHQ	9.20 (7.19)	9.45 (8.05)	9.14 (6.38)	0.2	0.846
PAM Score	66.53 (19.20)	65.49 (20.31)	67.49 (28.29)	− 0.47	0.638
Self-reported Adherence 7-Day	69.38 (32.58)	63.68 (34.91)	74.52 (29.81)	-1.5	0.138
Self-reported Adherence 30-Day	71.66 (25.29)	68.55 (27.18)	74.33 (23.60)	− 0.93	0.355
Medication Self-efficacy Score	4.47 (0.53)	4.46 (0.57)	4.47 (0.49)	− 0.125	0.901

### Intervention Effects

After the initial three-month intervention, 7-day adherence increased from 63.68 to 90.69% for those assigned to CPS, and 74.52–76.18% for those assigned to SMS. Overall Longitudinal Modeling with repeated measures every 3 months for the 12-month study demonstrated significant improvements in adherence for both groups (SMS change Chi-square = 41.03, *p* <.0001, CPS change Chi-square = 45.66, *p* <.0001), but CPS was significantly more effective than SMS as evidenced by time and group interaction (ES = 2.4359, SD = 1.1013, *p* = .027). For 30-day adherence at 3 months, CPS increased from 68.55 to 87.56%, and SMS increased from 74.33 to 84.87%. However, the longitudinal analysis did not demonstrate a significant difference between the two conditions over 12-months (*p* = .82) (see Table 2).

**Table 2:** Longitudinal model predicting medication adherence

	Baseline		3 Month		6 Month		9 Month		12 Month		Overall Longitudinal Model^a^			
	CPS	SMS	CPS	SMS	CPS	SMS	CPS	SMS	CPS	SMS	Estimate	Standard Error	Confidence Interval	*p*-value
Adherence 7-day														
N	38	42	29	34	22	33	24	29	21	27	**2.44**	**1.10**	**0.28**,** 4.59**	**0.027**
Mean	63.68	74.52	90.69	76.18	80	84.55	79.17	82.76	73.33	79.63				
SD	34.91	29.81	15.57	34.20	31.93	22.09	29.18	23.89	38.12	30.19				
Adherence 30-day														
N	31	36	27	30	21	31	23	27	18	25	0.17	0.76	-1.32, 1.66	0.82
Mean	68.55	74.33	87.56	84.87	85.48	82.13	78.39	83.74	83.94	80.60				
SD	27.18	23.60	20.35	22.73	21.14	20.08	27.92	21.33	22.73	28.73				

Controlling for Race/Ethnicity, Age, Gender Identity, Substance Use, Depression, Self-Efficacy for Medication, and Patient Activation

Analyzing changes in viral load was not feasible. While 69 of 83 participants had viral loads at baseline, only 43 had viral loads at 3 months, 35 at 6 months, 24 at 9 months, and 11 at 12 months. While the percentage of viral loads suppressed at < 200 went up in both CPS (58.8–89.5%) and SMS (62.9–87.5%) at 3-months and maintained throughout the study, we lacked power to reach significance. Lack of viral load data was attributed to difficulties during the COVID pandemic in accessing in-person phlebotomy and having a study population that was not clinic-based. Likewise, we did not find any differences in substance use across groups or over time.

As this was an adaptive intervention, we had planned to be able to evaluate those that failed the initial CPS and SMS arms (defined as adherence ≤ 80% of VL ≥ 200 at the 3-month visit). However, only 6/33 participants in the CPS arm failed the intervention, and only 8/36 youth in the SMS intervention failed the intervention at 3 months. Therefore, only descriptive data are presented, and analyses are limited to exploratory. Seven-day adherence > 80% between 3- and 6-month visits for the CPS plus Incentives (CPS-I) group changed from 0/3 participants to 4/4 participants (who provided data). At 9 months the CPS-I 7-day adherence > 80% was 3/5. Seven-day adherence > 80% for the SMS-I group changed from 2/7 participants to 4/8 between the 3- and 6-month visits and continued at 4/8 at the 9-month visit.

We conducted a secondary analysis of all participants successful on SMS or CPS at the 3-month visit (adherence > 80% or VL < 200). Those participants were randomized to either tapering to twice weekly calls (CPS-T) or tapering texts (SMS-T) or to standard of care (SOC). Please see Table 3 comparisons at each time point. Despite limited power for analyses, we did find several significant or near significant differences across groups. There was a significant improvement in adherence for the CPS-T compared to SOC at 6-months (*p* = .033). There was a significant improvement in self-reported adherence for SMS-T compared to SOC at 6-months (*p* = .006) but not at 9 months (*p* = .126). As we had limited power to determine differences, we also looked at combining all those with tapering (CPS-T + SMS-T) versus SOC and at 6-months (*p* = .001) and at 9 months (*p* = .117).

**Table 3:** Comparisons of achieving > 80% adherence in cellphone support tapered (CPS-T) and Text Messaging tapered (SMS-T) groups compared to Standard of Care (SOC) groups

Time point Comparison	Tapered Group (> 80% adherent/ participant count)	Standard of Care (> 80% adherent/ participant count)	*p*-values†
3-month	CPS-T (11/11)	SOC (24/29)	0.2824
6-month	CPS-T (8/8)	SOC (12/21)	**0.0332**
9-month	CPS-T (6/7)	SOC (13/18)	0.6372
3-month	SMS-T (12/13)	SOC (24/29)	0.6468
6-month	SMS-T (13/13)	SOC (12/21)	**0.0061**
9-month	SMS-T (11/11)	SOC (13/18)	0.1261
3-month	CPS-T + SMS-T (23/24)	SOC (24/29)	0.2044
6-month	CPS-T + SMS-T (21/21)	SOC (12/21)	**0.0013**
9-month	CPS-T + SMS-T (17/18)	SOC (13/18)	0.1774

† = Fischer’s Exact Test

## Discussion

In our sample of poorly adherent youth with baseline self-reported adherence ≤ 80% or viral load ≥ 200, we found significant improvements in self-reported adherence over the 12-month study for both CPS and SMS. While we lacked adequate viral load data to confirm these results, it is helpful that our percentage of participants with complete viral load suppression increased for both CPS and SMS. These improvements in both groups are similar to a pilot CPS study [10] and the randomized controlled trial of SMS [14]. Our results comparing CPS to SMS are more difficult to interpret. It is unclear why 7-day self-reported adherence was found to have a significant benefit for CPS over SMS over the 12-month study but was not found with 30-day self-report (although there was a higher percentage [19%] improvement in 30-day adherence in the CPS group and only 10.5% improvement in the SMS group). One might hypothesize that 7-day recall would be better than 30-day recall, but we have not seen this in previous youth adherence studies.

One important factor to note is the level of baseline nonadherence in this study and the pilot studies. In the CPS pilot study [10], baseline 30-day adherence was 32%, and 7-day self-reported adherence was 47%. The baseline 30-day adherence in Garofalo’s *Text Messaging* study (67%) was modest and similar to baseline 30-day self-reported adherence in the present study (72%) [8]. It is possible that in a more modestly nonadherent population (adherence levels 67–72%) CPS does not improve adherence more than the less intensive SMS intervention. CPS was highly effective for our extremely nonadherent population in the CPS pilot (baseline adherence only 32%), but the pilot study struggled with only about half adhering to the intervention. Future research can consider exploring these questions around treatment personalization of which intervention to begin with in clinical settings (answers we had hoped this study would answer). These questions of superiority will be difficult to answer because with our current, highly effective medications where most patients are on second generation integrase inhibitors (dolutegravir and bictegravir), durable viral suppression may only require 60–70% adherence due to long half-lives and medication potency. Future research might continue using adaptive treatment designs such as SMART, but also consider small-N designs given the challenge of recruiting youth with HIV to many trials.

The current study did inform several approaches to personalizing treatment within our target population towards better health outcomes. One possible way to utilize these interventions might be to start with SMS and transition to CPS for those continuing to fail viral suppression. Another approach might be to utilize CPS for those with severe nonadherence. One critical finding from our study was that we found the initial SMS and CPS were effective without incentives used in the CPS pilot, which will make implementation much more feasible.

Our adaptive trial had hoped to determine if sequentially adding incentives to participants who failed the first 3 months of CPS or SMS would be beneficial; however, due to low failure rates, only a few participants were randomized to CPS-I or SMS-I. We did note that over half the participants failing SMS and CPS did become adherent over the next 3–6 months suggesting a role for incentives (used exclusively in our pilot). Again, because of the availability of highly effective integrase inhibitors, the need for incentives should be uncommon.

The use of tapering CPS and SMS to twice weekly for an additional 3 months appears to be effective, though results should be interpreted with caution given the limited sample. All participants receiving tapering (21/21) at 6-months reported adherence > 80%, and 17/18 at 9-months maintained adherence > 80%. In the control arm only 12/21 participants maintained adherence at 6 months and 13/18 at 9 months. We hypothesize that having 6 months of intervention (3 months of daily followed by 3 months of tapering) is much better at maintaining adherence, even after the intervention ends. This is like our pilot data that utilized 6 months of daily CPS-I and demonstrated significant improvements of adherence for a full 6 months after the intervention ended.

### Limitations

Study limitations include small sample size reflecting difficulties in recruitment both before and during the COVID pandemic. As we were only able to recruit 83 participants (our original design was for 190 participants) we were unable to execute our SMART design as planned. While our study did include multiple randomizations, these multiple treatment pathways lacked the power to analyze. Therefore, our primary analyses reflect more of a traditional randomized clinical trial (i.e., first randomization). Analyses of dose and incentives were limited to exploratory. Difficulties in retention and obtaining viral load data limited our power to find significant differences between groups. It is unclear how relying on social media advertising to recruit our population impacted our findings compared to previous studies that used clinic-based populations. We note that communication of clinical teams with AFs was much more robust for those with clinic-based recruitment and implementation in the pilot study as compared to centralized implementation used in this study. It is useful that our study population tracked that of the HIV epidemic including a population of mostly young men of color who have sex with men and a majority from the Southern US, and perhaps this means our study population is more closely representative of youth in the US living with HIV.

In conclusion, our results are encouraging in that both SMS and CPS, both low-intensity, lower-cost interventions, improved adherence and did not require incentives for adherence to the intervention. In addition, this study was the first to test a centralized intervention platform in youth with HIV. Taken with our pilot intervention findings, this indicates implementation at both the clinic level and national level are feasible. Lastly, in both this study and the pilot of CPS with incentives, we demonstrate the durability of these mHealth interventions past the 6 months of intervention to the end of our one-year trial. How to sequence these two efficacious interventions remains unanswered although our data suggest that 7-day self-reported adherence was better with CPS and might be initiated first for highly nonadherent patients, those at more imminent risk of AIDS-related symptoms, or those with depression or substance misuse.
